# News and Miscellany

**Published:** 1881-09-20

**Authors:** 


### *NEWS AND MISCELLANY.*

The American Public Health Association will hold its ninth session in Savannah, November 19th.

*Pancreopepsine.*—See Warner & Co.'s new advertisement of Liquid Pancreopeptine.

NICE Chemical preparations are being put up by George J Howard & Bro., wholesale and retail druggists on Broad street. See their advertisement.

*The Southern Medical College* will commence its Third Annual Session on October 13th. Both hospital and clinical advantages will be furnished the student the coming session, and in all respects the facilities for instruction will be of a high order.

*Celerina.*—This preparation is highly spoken of and seems to be growing rapidly in the confidence of the profession. See the advertisement in this Journal. It is especially recommended in the opium habit, and is adapted to low states of the nervous system from any cause.

*Surgical Instruments.*——We are informed that the house of A. L. Hernstein, of New York, will soon establish a branch house or Southern Depot for Surgical Instruments in Atlanta, so that hereafter physicians may procure any instrument in the Surgical line without the necessity and delay of ordering from New York.

